# An Overlapping Case of Miller Fisher Syndrome, Bickerstaff's Encephalitis, and the ASMAN Variant of Guillain-Barre Syndrome

**DOI:** 10.1155/2016/1596850

**Published:** 2016-11-06

**Authors:** E. J. Pegg, S. K. Chhetri, U. G. Lekwuwa, T. Majeed

**Affiliations:** Department of Neurosciences, Royal Preston Hospital, Preston, UK

## Abstract

A 56-year-old man presented with a 3-day history of progressive tingling of the hands, unsteadiness, and diplopia. He was initially diagnosed clinically with Miller Fisher Syndrome (MFS) but later developed limb weakness consistent with Guillain-Barre Syndrome (GBS) and subsequently reduced consciousness consistent with Bickerstaff's brainstem encephalitis (BBE). Neurophysiology revealed an axonal motor and sensory neuropathy, in keeping with the Acute Motor and Sensory Axonal Neuropathy (AMSAN) variant of GBS. We believe that our patient had an MFS-AMSAN-BBE overlap syndrome. This is supported by his glycolipid antibody profile with high titres of anti-GQ1b IgG antibody and anti-GD1a IgG antibody. Anti-GQ1b antibodies are frequently found in both MFS and BBE and the anti-GD1a antibody is associated with axonal forms of GBS. Overlapping cases of MFS and BBE are well described, and because the same antibody is often found in both conditions, it is thought that they share a common autoimmune mechanism. BBE has also been previously reported in association with GBS lending support that it also lies on the same spectrum. This overlapping case of ASMAN variant of GBS, MFS, and BBE provides further support that these conditions are part of the same spectrum.

## 1. Introduction

MFS is a triad of ophthalmoplegia, ataxia, and areflexia and is associated with antibodies to anti-GQ1b ganglioside. It is frequently seen in association with GBS and therefore is thought to be part of the same spectrum of illness. BBE includes signs and symptoms of ophthalmoplegia, ataxia, and reduced consciousness. It is also associated with anti-GQ1b antibody and can coexist with GBS. GBS can be further categorised according to neurophysiology findings. The AMSAN form of GBS is associated with anti-GD1a antibody.

We describe a case of a patient with features of an overlap syndrome of MFS-AMSAN-BBE, supported by a glycolipid antibody profile of high titres of anti-GQ1b IgG antibody and anti-GD1a IgG antibody. This case lends support to the hypothesis that these conditions are part of the same spectrum and share a common autoimmune mechanism.

## 2. Case Report

A 56-year-old Caucasian man with no past medical history of note presented with a 3-day history of progressive tingling of the hands, unsteadiness, and diplopia. He recalled a mild cough 2 weeks prior to the onset but did not have any other infective symptoms.

On examination he had complete ophthalmoplegia, facial diplegia, bulbar dysarthria, mild bilateral shoulder abduction weakness, bilateral upper limb ataxia, generalised areflexia, impaired vibration sense to both clavicles, and a broad based ataxic gait. Initial cerebrospinal fluid (CSF) studies were normal. A clinical diagnosis of Miller Fisher Variant (MFS) of Guillain-Barre Syndrome (GBS) was considered and he received intravenous immunoglobulin (IVIG) therapy. The diagnosis was subsequently supported by high titres of anti-GQ1b IgG antibody. Anti-GD1a IgG was also positive.

The following week, a generalised limb weakness developed. Repeat CSF studies showed albuminocytologic dissociation with white cell count of less than 1 × 10^6^/L and protein of 1.58 g/L (normal range). CSF glucose was 5 mmol/L and serum glucose 3.7 mmol/L. Nerve conduction studies (NCS) undertaken 17 days after symptom onset demonstrated background generalised axonal motor and sensory polyneuropathy. On concentric needle electromyography, there was evidence of associated peripheral nerve hyperexcitability with increased insertional activity, fasciculation potentials, and myokymic discharges in several limb muscles. In view of the continued deterioration, a second course of IVIG was given, 4 weeks after the initial course.

The clinical condition remained fairly static following the 2nd course of IVIG. However, 6 weeks after the initial symptom onset, there was a significant drop in his conscious level raising concerns about BBE. Proximal and distal limb wasting had become apparent in keeping with evolution of ASMAN. He was subsequently transferred to the intensive care unit for closer monitoring where he received plasma exchange but did not require respiratory support. Electroencephalography (EEG) revealed slow background activity but no epileptiform discharges. Magnetic resonance imaging (MRI) of the brain was normal. Metabolic and medication causes of encephalopathy were excluded. His conscious level improved 4 days later and he returned to the ward.

Over the next 3 months, his symptoms gradually improved and currently he has very mild facial weakness and uses a frame to walk.

## 3. Discussion

Our patient was initially diagnosed with MFS given the classic triad of ophthalmoplegia, areflexia, and ataxia but he later developed limb weakness consistent with GBS. NCS revealed an axonal motor and sensory neuropathy which was therefore consistent with the Acute Motor and Sensory Axonal Neuropathy (AMSAN) variant of GBS. The subsequent impairment in consciousness/encephalopathy, together with features of MFS and anti-GQ1b antibody, is consistent with Bickerstaff's brainstem encephalitis (BBE) as outlined in the diagnostic criteria [1]. We therefore believe that our patient had an MFS-AMSAN-BBE overlap syndrome. A normal MRI and absence of pyramidal signs should not dissuade one from the diagnosis of BBE as only 23−30% of patients have an abnormal MRI; similarly brisk reflexes and Babinski response are found in 13–34% and 40−43% of the cases, respectively [1, 2].

Anti-GQ1b antibodies are frequently found in both MFS and BBE [3] and the anti-GD1a antibody is reported to be associated with axonal forms of GBS [4]. Overlapping cases of MFS and BBE are well described in the literature, and because the same antibody is often found in both conditions, it is thought that they are part of the same spectrum with a common autoimmune mechanism [3]. BBE has also been previously reported to overlap with GBS lending support that GBS is also on the same clinical spectrum. In a study of 62 patients who had been diagnosed with BBE, almost 60% had significant limb weakness. 24 of the 37 patients had NCS which revealed axonal degeneration in 50% and demyelination in 8% [2]. To the best of our knowledge, overlapping MFS-BBE-GBS has only been previously reported in 4 cases (Table 1), all with slightly differing antibody profiles.

## 4. Conclusion

This overlapping case of ASMAN variant of GBS, MFS, and BBE provides further support that these conditions are part of the same spectrum and share a common autoimmune mechanism.

## Figures and Tables

**Table 1:** Reported cases of overlapping MFS-BBE-GBS.

Author	Antibody	NCS
Fujii et al. 2012 [5]	GM1, GD1a, GQ1b, GT1a	Axonal motor and sensory polyneuropathy
Arai et al. 2002 [6]	GQ1b	Not reported
Han et al. 2012 [7]	Negative	Axonal motor and sensory polyneuropathy
Stevenson et al. 2003 [8]	GM1	Axonal motor and sensory polyneuropathy
Pegg et al. 2016	GD1a, GQ1b	Axonal motor and sensory polyneuropathy
